# ADVANCING AGING IN THE RIGHT PLACE THROUGH MULTIPLE ECOLOGICAL DOMAINS

**DOI:** 10.1093/geroni/igae098.0290

**Published:** 2024-12-31

**Authors:** Anthony Traver, Amanda Lehning

**Affiliations:** Chair; Discussant

## Abstract

Individuals experiencing housing insecurity encounter service gaps and environmental inadequacies that compromise their health, social inclusion, and resilience in later life. The Aging in the Right Place (AIRP) framework provides a model for recognizing and addressing these gaps and inadequacies across multiple ecological domains. This symposium brings together five papers to examine the interplay of housing, community context, and AIRP. The first paper uses photovoice and qualitative geospatial methods to investigate the social challenges of aging faced by older people experiencing homelessness (PEH). The second paper analyzes interviews to examine how the usage and perceptions of transportation resources differ between younger and older PEH. The third paper uses data from the National Health and Aging Trends Study to examine the influence of various housing typologies on participation in social and physical activities among older adults with functional limitations. The fourth paper profiles the implementation of emergency preparedness strategies by a senior housing advocacy group to enhance the resilience of affordable housing communities. Finally, the fifth paper presents results from a randomized controlled trial to demonstrate the effect of guaranteed basic income on housing access among older PEH. The symposium will conclude with a discussion of innovative community-level strategies to enhance health, inclusion, and resilience among adults who are attempting to age in their right place while economically and socially marginalized.

